# Predicting hospital admission and discharge with symptom or function scores in patients with schizophrenia: pooled analysis of a clinical trial extension

**DOI:** 10.1186/1744-859X-9-24

**Published:** 2010-06-02

**Authors:** Chris M Kozma, Riad G Dirani, Carla M Canuso, Lian Mao

**Affiliations:** 1 University of South Carolina, Columbia, SC, USA; 2 Ortho-McNeil Janssen Scientific Affairs, LLC, Titusville, NJ, USA

## Abstract

**Background:**

The purpose of this analysis was to evaluate relationships between hospital admission or discharge and scores for symptom or functioning in patients with schizophrenia.

**Methods:**

Data were from three 52-week open-label extensions of the double-blind pivotal trials of paliperidone extended-release (ER). Symptoms and patient function were measured every 4 weeks using the Personal and Social Performance (PSP) scale and the Positive and Negative Syndrome Scale (PANSS). The intent-to-treat analysis set was defined as open-label patients who had at least one post-baseline PSP and PANSS measurement. Time until first hospitalization was evaluated using the Cox proportional hazard model with categorical time-dependent measures for the PSP (1 to 30, 31 to 70, 71 to 100) or PANSS (< 75, ≥ 75 to < 95, ≥ 95), as well as age, gender, schizophrenia duration, and country. Similar analyses were performed for time to discharge.

**Results:**

Of the 1,077 enrolled patients, 1,028 (95.5%) met study criteria; of these, 382 (37.2%) were hospitalized at open-label baseline. Compared with patients with PSP ≥ 71 group, the hazard for new hospitalization was 8.351 times greater (*P *= 0.0001) for patients with the poorest functioning (PSP 1 to 30) and 1.977 times greater (*P *= 0.0295) for patients with PSP of 31-70 compared to the ≥ 71 group. The hazard for new hospitalization was 5.457 times greater (*P *< 0.0001) for patients PANSS ≥ 95 and 2.316 times greater (*P *= 0.0027) for the ≥ 75 to < 95 group compared with the < 75 group. For patients hospitalized at baseline, the PANSS ≥ 95 patients had a discharge hazard that was 0.456 times lower than for the < 75 patients (*P *< 0.0001). The hazard for discharge was 0.646 times lower (*P = *0.0012) for the PANSS ≥ 75 to < 95 group compared with the < 75 group. A patient's country was a significant predictor variable, with US patients being admitted and discharged faster.

**Conclusions:**

Better functioning or being less symptomatic is associated with reduced risk for hospitalization and greater chance for early discharge. Treatments or programs that reduce symptoms or improve function decrease the risk of hospitalization in community patients or increase the chance of discharge for hospitalized patients.

## Background

Much of the clinical trial literature in schizophrenia focuses on symptom improvement. The Positive and Negative Syndrome Scale (PANSS) [1] is a standard assessment in many trials. In a PubMed literature search, PANSS has been cited or used in research more than 250 times [2]. Given the chronic nature of schizophrenia and need for maintenance therapy, most drugs are evaluated for their efficacy in improving acute symptoms (such as delusions and hallucinations) as well as preventing recurrence. For example, compared with placebo, paliperidone extended release (ER), has been shown to delay time to recurrence (23 days vs 83 days, 25% quartile, respectively, *P *= 0.005) and was associated with lower rates of recurrence 53% vs 25%, respectively). In that study, recurrence was defined using PANSS score change, psychiatric hospitalization, self-injury, and suicidal or violent behavior [3].

Function scales are fundamentally different than symptom scales in the domain being assessed because they measure behaviors such as self-care or social interaction [4]. These behaviors can exist even if the person is experiencing symptoms such as delusions or hallucinations. (For a discussion of the tools and role of functional assessment in research, see the white paper summarizing the conclusions from a National Institute of Mental Health workshop [5].) The Personal and Social Performance (PSP) scale is an example of a function scale useful in many conditions, including schizophrenia [6]. It and related versions of the instrument have been used for more than a decade in multiple studies [7]. Pearson correlation coefficient for the association between baseline PSP and PANSS total scores was -0.32 for subjects assessed by the same rater and -0.29 for subjects assessed by different raters, suggesting low overlap in measurement constructs between the PANSS and PSP [8].

The Remission in Schizophrenia Working Group [9] has reviewed various symptom and function scales and their relationship to the state of remission. Using the PANSS scale, they recommended a score of 3 (mild) or less on the 7-point scale for 6 or more months to be consistent with symptomatic remission of the disease. As additional experience is gained, symptom scores may be benchmarked to different levels of function and/or hospitalization. Studies have been published validating function scales such as the PSP for use in research [8]; however the Working Group did not make a remission definition based on functional scores at this time.

**Figure 1:**
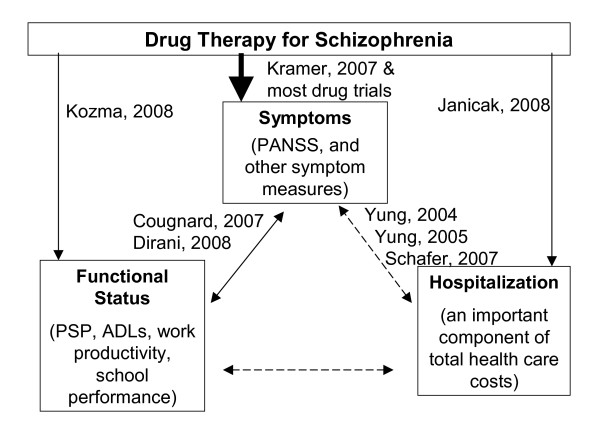
**A partial listing of studies that have reported the relationship between symptoms, functional status, and hospitalization and how those outcomes are influenced by drug therapy**. The dotted line relationships are the focus of this research.

As a means of focusing on the concept of remission, the present research will focus on hospitalization (an observable event), and the relationship to scores for symptoms or function. Figure 1 is provided as a partial overview of the relationships between the three concepts being assessed in this research. The use of drug treatment to achieve symptom improvement is often well studied but the relationships between hospitalization and the concepts of symptoms or functioning are less well understood. Although appropriate drug therapy is likely to have positive effects on symptoms, function, and hospitalization, research (such as the present work) to better understand the complexity and magnitude of the relationships among the various types of outcomes is needed.

Several examples of research of these relationships shown in Figure 1 can be identified in the published literature. The relationship between symptoms and functional status as measured in a clinical trial using commonly accepted instruments was reported by Dirani *et al*. [10] The relationship between symptoms and hospitalization or psychotic events was described by Yung *et al*. in Australia [11,12] and Schefer *et al*. in Austria [13], who developed methods to predict a psychosis event (often hospitalized) based on symptoms and other historical factors. Other researchers focused on the functional status or hospitalization outcomes in schizophrenia. Cougnard *et al*. found that the median time delay between the onset of schizophrenia symptoms (diagnosis) and the first request for disability status was 4 years in France [14].

The relationship between treatment with paliperidone ER and reduced rates of hospitalization were reported in an open label before/after evaluation by Janicak *et al*. [15]. Using a similar design, Kozma *et al*. reported that after extended treatment with paliperidone ER the percentage of patients who were employed almost doubled [16]. These reports evaluated five of the six relationships shown in Figure 1, adding to our understanding of these relationships. This is not meant to be a complete review of the research in this field, only illustrative of the studies of these constructs.

Although the research to date has been helpful, a clear understanding of the relationship between hospitalization and symptoms or function continues to need further elucidation. The purpose of this analysis was to investigate if the understanding of the dynamics in Figure 1 could be enhanced through an investigation of the relationships between hospitalization and symptoms or function.

## Methods

### Data acquisition

Data were pooled from three 52-week open-label extensions of the 6-week, double-blind, pivotal trials of paliperidone ER conducted in men and women 18 years of age or older with a diagnosis of schizophrenia. These three pooled trials shared a similar design, including a 12-month open-label extension. The results of these trials have been published separately [17-20]. The research was conducted in compliance with the Helsinki Declaration, and approved by the local institutional review board governing each research site.

Inclusion criteria for the double-blind portion of the trials included age of 18 years or older, diagnosis of schizophrenia according to *Diagnostic and Statistical Manual of Mental Disorders, fourth edition *(DSM-IV) criteria at least 1 year prior to the screening visit, an acute episode with a PANSS score between 70 and 120 (moderate to markedly ill), agreement with voluntary hospitalization for at least 14 days, compliance with self-administered medication or consistent support, and informed consent. Exclusion criteria included a DSM-IV axis I diagnosis for any condition other than schizophrenia or a DSM-IV diagnosis of substance abuse within 6 months before screening (not including nicotine and caffeine addiction). Additional inclusion and exclusion criteria are presented in the previous reports from the randomized blinded phase of these trials [17-20]. Patients were eligible for the open-label extension if they completed the double-blind phase or discontinued the double-blind phase owing to lack of efficacy after at least 21 days of treatment, signed consent for the open-label phase, and the investigator agreed that the open-label phase was in their best interest.

Data in the open-label phase of the three trials were collected under similar protocols. Subjects received flexibly dosed paliperidone ER (3, 6, 9, 12, and 15 mg; the 15 mg dose was available in only one trial) administered once daily for 52 weeks. Study visits occurred weekly for the first 4 weeks and every 4 weeks thereafter. The intent-to-treat analysis set was defined as open-label patients who had at least one post-baseline PSP and PANSS measurement.

### Definitions

#### PANSS

The development of the PANSS was published in 1987 [1]. Schizophrenia symptoms such as delusions, hallucinations, blunted affect, social and emotional withdrawal, and so on were assessed using the 30-item PANSS scale. PANSS scores were summed across the 30 items to derive a total score. Each item was rated on a scale of 1 (absent) to 7 (extreme). An average rating of 3 (mild) or a total score of 90 was proposed by the Working Group as being remission [9]. This study used the total PANSS score divided into three categories: high symptomatology (≥ 95), medium symptomatology (≥ 75 and < 95), and low symptomatology (< 75) patient groups, respectively. These cut-off points have been used in previous studies, and they also correspond to points slightly below and above the definition of remission as proposed by the Working Group (as a form of a sensitivity analysis).

#### PSP

The development of the PSP was initially published in 2000, and was previously called the SOFAS (Social and Occupational Functioning Assessment Scale) [6]. It assesses the degree of difficulty a subject exhibits over a 1-month period within four domains of behavior: socially useful activities, personal and social relationships, self-care, and disturbing and aggressive behavior. The results of the assessment were converted to a numerical score following the PSP scoring guidelines. The score ranges from 1 to 100. Subjects with scores from 71 to 100 have a mild degree of difficulty; from 31 to 70, varying levels of disability; and from 1 to 30 are functioning so poorly as to require intensive support and supervision. These cut-off points of 30 and 70 have been used in other studies, and they are derived directly from the scoring of the instrument. Scores above 71 generally represent patients who have 'mild' ratings in the majority of areas, scores from 31-70 occur when the ratings are 'moderate' in the majority of areas, and below 30 occurs when the ratings are 'severe' in the majority of areas.

#### Hospitalization

Time until first hospitalization for 'psychotic disease' was used for patients who were not hospitalized at the start of the open-label phase. Time until first discharge was used for patients who were hospitalized at the start of the open-label phase.

#### Analysis

The design for the current analysis was a single group evaluation of associations between hospital admission or discharge and symptom or function scores. Patients who qualified for the open-label extension of the three randomized clinical trials and who had usable scale scores and valid hospitalization dates were included in the analysis. All patients included in the analysis were treated with paliperidone ER. The observation period was from the start of the open-label phase until day 351 (earliest possible start of the last treatment window).

Categorical variables were summarized using frequencies and percentages. Continuous measures were summarized with mean, standard deviation, minimum, maximum, and median. Relationships between risk of hospitalization or discharge, symptoms or function were conducted using Cox proportional hazard regression models. Models were evaluated for time until first psychosis-related hospitalization (for patients who were not hospitalized at the start of the observation period) of the open-label phase. Explanatory variables of the Cox model included double-blind treatment, age, gender, duration of illness, study country (US vs non-US), as well as a time-dependent PSP score (1 to 30, 31 to 70, ≥ 71) or time-dependent PANSS total score (< 75, 75 to 94, ≥ 95). Indicator variables were created for the 3 PSP and 3 PANSS categories, with the reference categories being PSP ≥ 71 and PANSS < 75. The PSP or PANSS score measured at the assessment prior to the hospitalization (or discharge) were used in the Cox model. Similar Cox models were used for time until first hospital discharge (for patients hospitalized at baseline).

SAS version 9.1 was used for all analyses (SAS, Chicago, IL, USA). All tests were two tailed and were conducted at the 5% significance level.

## Results

### Sample

The combined open-label, intent-to-treat sample included 1,077 patients from the 1,665 patients who enrolled in the double-blind randomized portion of the clinical trials. The intent to treat analysis set was defined as open-label patients who had at least one post-baseline PSP and PANSS measurement. Of the 1,077 patients who continued to the open-label phase, 1,028 were used in this analysis. Less than 5% (49 patients) were excluded for reasons that included no open-label PSP scores (n = 31), 1 day or less in the open-label phase (n = 4), or invalid hospital dates (n = 14). Of the 1,028 patients included in the study, 646 (62.8%) were not hospitalized for psychosis at the start of the open-label period whereas 382 (37.2%) were hospitalized for psychosis at the same point.

### Demographics and patient characteristics

The mean age was 37.3 ± SD 10.9 years for the group that was hospitalized at the start of the open-label phase and 37.8 ± SD 11.0 years for the group that was not hospitalized when the open-label phase began. Approximately 60% were male, and almost half of each sample had duration of schizophrenia of 10 or more years. In all, 91% of patients hospitalized at the start of the open-label phase and 69% of patients not hospitalized at that point were enrolled at sites in countries other than the US. Approximately 50% of the sample discontinued the open-label study by week 52. The survival analysis used data up to the point of study discontinuation (Table 1).

**Table 1:** Baseline demographic and patient characteristics

Parameter	Statistics	
	
	Not hospitalized at start of the open-label phase	Hospitalized at start of the open-label phase
Age, years		
n	646	382
Mean (SD)	37.8 (11.0)	37.3 (10.9)
Median (range)	38.0 (18.0; 76.0)	36.0 (18.0; 66.0)
		
Age groups, n (%)		
18 to < 35	278 (43.0)	169 (44.2)
35 to < 55	322 (49.8)	191 (50.0)
≥ 55	46 (7.1)	22 (5.8)
		
Gender, n (%)		
Female	254 (39.3)	165 (43.2)
Male	392 (60.7)	217 (56.8)
		
Schizophrenia duration, n (%)		
0 to < 4 years	166 (25.7)	92 (24.1)
4 to < 10 years	189 (29.3)	121 (31.7)
≥ 10 years (includes 2 missing)	291 (45.0)	168 (44.0)
		
Study country, n (%)		
		
US	198 (30.7)	34 (8.9)
Non-US	448 (69.3)	348 (91.1)
		
Remaining in trial, n (%)		
Week 1	646 (100.0)	382 (100.0)
Week 4	646 (100.0)	382 (100.0)
Week 8	563 (87.2)	315 (82.5)
Week 12	522 (80.8)	286 (74.9)
Week 16	490 (75.9)	263 (68.8)
Week 20	473 (73.2)	252 (66.0)
Week 24	446 (69.0)	242 (63.4)
Week 28	414 (64.1)	229 (59.9)
Week 32	400 (61.9)	222 (58.1)
Week 36	379 (58.70	210 (55.0)
Week 40	365 (56.5)	202 (52.9)
Week 44	354 (54.8)	193 (50.5)
Week 48	335 (51.9)	189 (49.5)
Week 52	319 (49.4)	187 (49.0)

### Hospitalization/discharge data and PANSS and PSP scores

Of the 646 patients who were not hospitalized at the start of the open-label phase, 67 (10.4%) had at least 1 hospitalization during an average of 239.2 (SD 131.8) days of observation. The average time until the initial open-label hospitalization was 92.1 (SD 85.1; median 63.0) days. Of the 382 patients who were hospitalized at the start of the open-label phase, 299 (78.3%) were discharged. The average time to discharge was 37.0 (SD 42.4; median 25.0) days.

**Figure 2:**
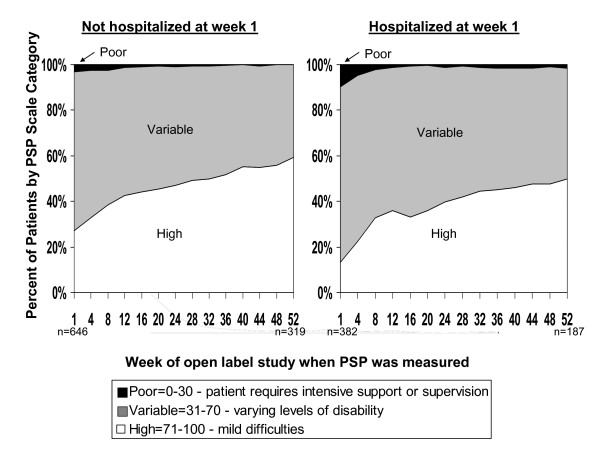
**Percentage of patients by Personal and Social Performance (PSP) scale category by week of PSP administration and hospitalization status at week 1 (the start of the open-label phase)**.

**Figure 3:**
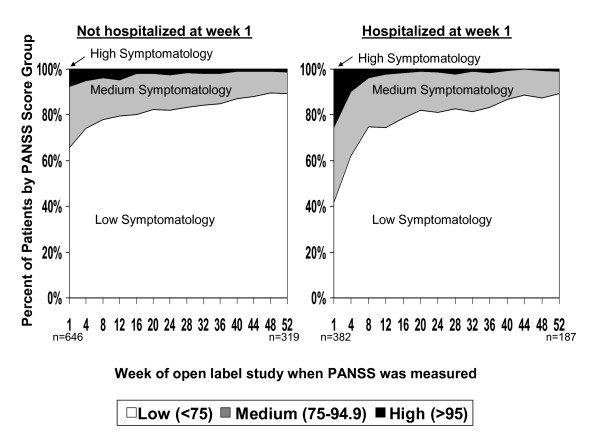
**Percentage of patients by Positive And Negative Syndrome Scale (PANSS) score category by week of PANSS administration and hospitalization status at week 1 (the start of the open-label phase)**.

The mean PSP and PANSS scores improved over time in both groups (Figures 2 and 3) for two reasons. In general, the longer an individual stayed in the study, the more his or her symptom and function scores improved. In addition, patients who discontinued the study had higher average symptom and lower function scores at baseline. Both of these factors resulted in the symptom and functional improvements over time for the cohorts. Patients who discontinued had a mean PSP score of 56.8 (SD 15.1) versus 60.6 (SD 15.4) for those who did not discontinue (*P *< 0.0001). Patients who discontinued had a mean PANSS score of 75.1 (SD 21.1) versus 70.3 (SD 19.9) for those who did not discontinue (*P *< 0.0001).

### Survival analysis results

Survival analyses of the relationships between risk of hospitalization or discharge, and function (PSP), or symptoms (PANSS) were conducted using four different Cox proportional hazard regression models, outlined below.

Model A: predicting hazard for hospital admission with PSP (poor and variable functioning with high functioning as the reference). Model B: predicting hazard for hospital admission with PANSS (high and medium symptomatology with low symptomatology as the reference). Model C: predicting hazard for hospital discharge with PSP (poor and variable functioning with high functioning as the reference). Model D: predicting hazard for hospital discharge with PANSS (high and medium symptomatology with low symptomatology as the reference).

The four models were evaluated for time until first psychosis-related hospitalization (for the patient group that was not hospitalized at the week 1 assessment) and for time until first hospital discharge (for the patient group hospitalized at the week 1 assessment) of the open-label phase.

**Figure 4:**
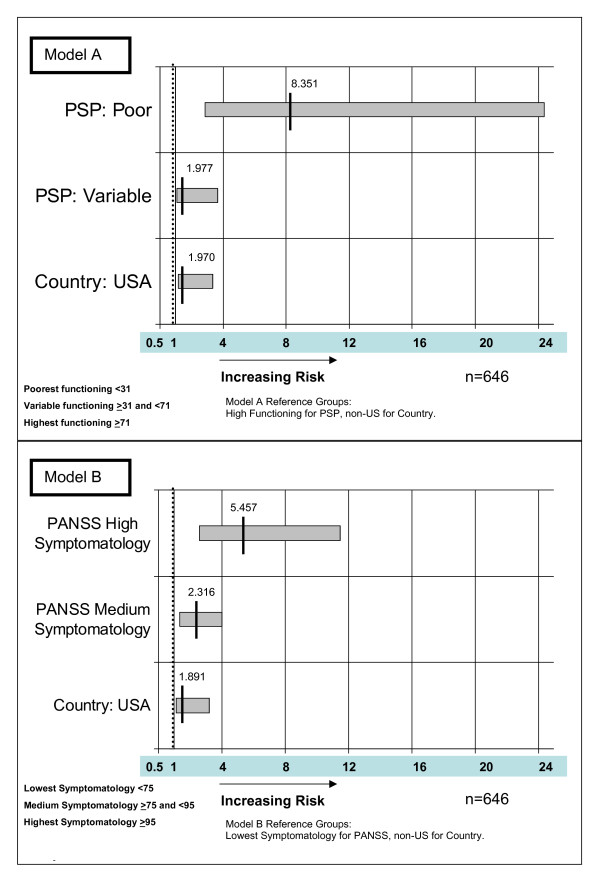
**Hazard ratios for significant variables in the two Cox proportional hazard regression models predicting hazard (risk) of hospitalization using Personal and Social Performance (PSP) scale (model A) and Positive And Negative Syndrome Scale (PANSS) (model B)**.

**Figure 5:**
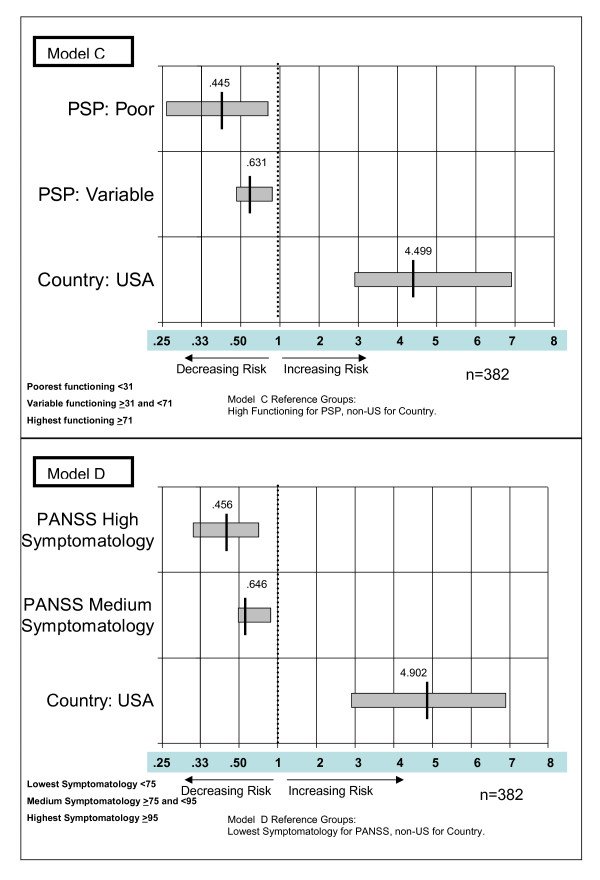
**Hazard ratios for significant variables in the two Cox proportional hazard regression models predicting hazard (chance) of hospital discharge using Personal and Social Performance (PSP) scale (model C) and Positive And Negative Syndrome Scale (PANSS) (model D)**.

The two categorical PSP and PANSS variables and the indicator of whether the patient was at a US or non-US site were significant in both of the two hospitalization models (Figure 4) and the two discharge models (Figure 5). Schizophrenia duration was significant in only the two 'time-until-discharge' models. Age and gender were not predictive of hospitalization or discharge in any of the four models.

### Predicting future hospital admissions

As shown in Figure 4, model A, for the patients not hospitalized at the start of the open-label phase, those with the lowest functioning (PSP 1 to 30) had a hazard for hospitalization that was 8.351 times greater (*P *= 0.0001; 95% CI 2.860 to 24.383) than patients with the highest functioning (PSP 71 to 100). Patients in the middle functioning group (PSP 31 to 70; 'varying levels of difficulty') had a hazard for hospitalization that was 1.977 times greater (*P *= 0.0295; 95% CI 1.070 to 3.652) than patients with the highest functioning (PSP 71 to 100). In model A, US sites had a hazard for hospitalization that was 1.970 times greater (*P *= 0.0115; 95% CI 1.165 to 3.331) than for non-US sites.

As shown in Figure 4, model B, the patients in the highest symptom score group (PANSS ≥ 95) had a hazard for hospitalization that was 5.457 times greater (*P *< 0.0001; 95% CI 2.597 to 11.466) than for patients in the lowest symptom score group (PANSS < 75). Patients in the middle symptom score group (PANSS 75 to < 95) had a hazard for hospitalization that was 2.316 times greater (*P *< 0.0027; 95% CI 1.338 to 4.009) than for patients with the lowest scores (PANSS < 75). In Model B, US sites had a hazard for hospitalization that was 1.891 times greater (*P *= 0.0176; 95% CI 1.117 to 3.200) than for non-US sites (Figure 4).

### Predicting future hospital discharge

The Cox proportional hazard regression model used the function score (PSP) or symptom scores (PANSS), and other covariates of the patients who were hospitalized on the day of their week 1 assessment in the open-label phase to predict the hazard for discharge from the hospital. As shown in Figure 5, model C, patients with the lowest functioning (PSP 1 to 30) had a hazard for discharge that was 0.445 times lower (*P *= 0.0039 (95% CI 0.257 to 0.771) than that for those with the highest functioning (PSP 71 to 100). This indicates that patients with low function scores are less than half as likely to be discharged compared with those having function scores between 71 and 100 (inclusive). Patients in the middle group (PSP 31 to 70; 'varying levels of difficulty') had a hazard for hospitalization that was 0.631 times lower (*P *= 0.0013; 95% CI 0.476 to 0.836) than patients with the highest functioning (PSP 71 to 100). US sites had a hazard for discharge that was 4.499 times greater (*P *< 0.0001; 95% CI 2.904 to 6.970) than for non-US sites.

It should be noted that hazard ratios in the range 0 to 1 indicate a decrease in risk for the event. For this 0-1 range to be visually proportional to the >1 range in the figure, the values less than 1 are graphed on a scale proportional to the inverse of the hazard rate; that is, a hazard score of 0.1 indicates the event is 10 times less likely to occur (1/0.1 = 10).

As shown in Figure 5 model D, patients in the highest symptom score group (PANSS ≥ 95) had a hazard for discharge that was 0.456 times lower (*P *< 0.0001; 95% CI 0.313 to 0.664) than that for those in the lowest symptom score group (PANSS < 75). This indicates that patients with high PANSS scores were less than half as likely to be discharged at any time than the patients with the low PANSS scores < 75. Patients in the middle symptom score group (PANSS ≥ 75 to < 95) had a hazard for discharge that was 0.646 times lower (*P *= 0.0012; 95% CI 0.496 to 0.841) than for patients with the lowest symptom scores (PANSS < 75). US sites had a hazard for discharge that was 4.902 (*P *< 0.0001; 95% CI 2.904 to 6.970) times greater than for non-US sites. This indicates that US sites discharged hospitalized schizophrenia patients at nearly five times the rate of non-US sites.

## Discussion

This analysis demonstrates that the validated functional status measure (PSP) and the widely used symptom assessment tool (PANSS) may be useful for identifying patients who are at increased risk of a mental health hospitalization. In the 1-year follow-up periods in these clinical trials, the assessments were performed monthly. In clinical practice, such assessments can be conducted to determine which patients are in need of additional support, monitoring, or treatment adjustment. If the same tools are used in the inpatient setting, they may help to identify which patients are ready for discharge. As experience is gained using symptom and functioning instruments, their use in remission criteria and guidelines may increase.

Other social and environmental factors such as stable housing and support networks may delay discharge or accelerate admission. These were not measured in the study and therefore could not be included in the predictive model. As with many forms of health care utilization, the threshold for admission and discharge may vary by country. Controlling for symptoms and function scores, this analysis indicated that patients in the US are more likely to be admitted faster and also more likely to be discharged faster (sometimes called 'the revolving door').

Interpretation of the Cox proportional hazard regression results should be based on the characteristics of this study population. There was a higher dropout rate among patients with higher symptoms and lower function scores at baseline. Although the study followed these patients to the point of discontinuation, the impact on hospitalization after disenrollment is unknown. Results could vary in other populations.

Given our findings, use of either function or symptom tools in the ambulatory setting is a good predictor of a future hospitalization. If identifying high-risk patients enables the clinician to prevent hospitalization or other negative outcomes, schizophrenia morbidity can be decreased and the direct and indirect costs of these diseases can potentially be lowered. Such assessment tools might give clinicians a better understanding of the impact of treatment on symptomatology, functional status, and health care resource use (hospitalization).

## Conclusions

Being more symptomatic or having poorer function appears predictive of hospitalization. For those already admitted, being symptomatic or having poor function is associated with a greater risk of not being discharged. Increase use of functional and symptom measurement tools in clinical practice is supported. Treatments or programs that reduce symptoms or improve function are likely to decrease the risk of hospitalization in community patients or increase the chance of discharge for hospitalized patients.

## Competing interests

Supported by funding from Ortho-McNeil Janssen Scientific Affairs, LLC. RD, LM and CC are employees of Ortho-McNeil Janssen Scientific Affairs, LLC. CK was contracted by Ortho-McNeil Janssen Scientific Affairs to perform the statistical analysis

## Authors' contributions

CMK contributed to design of the analysis, execution of the statistical analysis, interpretation of the data, and final approval of the manuscript. RD contributed to the design of the analysis, interpretation of the data, decision to publish, writing/editing of the text, and final approval of the manuscript. LM contributed to design of the analysis, execution of the statistical analysis, interpretation of the data, and final approval of the manuscript. CC contributed to the design of the analysis, interpretation of the data, decision to publish, writing/editing of the text, and final approval of the manuscript.
